# Effect of Cr, Mo and Al on Microstructure, Abrasive Wear and Corrosion Resistance of Ni-Mn-Cu Cast Iron

**DOI:** 10.3390/ma12213500

**Published:** 2019-10-25

**Authors:** Daniel Medyński, Bartłomiej Samociuk, Andrzej Janus, Jacek Chęcmanowski

**Affiliations:** 1 Faculty of Technical and Economic Sciences, Witelon State University of Applied Science in Legnica, Sejmowa 5A, 59-220 Legnica, Poland; 2 Department of Foundry Engineering, Plastics and Automation, Wroclaw University of Technology, Smoluchowskiego 25, 50-372 Wroclaw, Poland; bartlomiej.samociuk@pwr.edu.pl (B.S.); andrzej.janus@pwr.edu.pl (A.J.); 3 Department of Advanced Materials Technologies, Wroclaw University of Technology, Smoluchowskiego 25, 50-372 Wroclaw, Poland; jacek.checmanowski@pwr.edu.pl

**Keywords:** abrasive wear, austenitic cast iron, austenitic transformation, corrosion resistance, Ni-Mn-Cu cast iron

## Abstract

Results of a study on influence of Cr, Mo and Al on the microstructure, abrasive wear and corrosion resistance of Ni-Mn-Cu cast iron in the as-cast and heat-treated conditions are presented. Because of the chilling effect of first two elements (tendency to create hard spots), graphitising Al was added to the alloys, with the highest concentration of Cr and Mo. All castings in the as-cast condition showed an austenitic matrix, guaranteeing good machinability. Heat treatment of raw castings, consisting in annealing at 500 °C for 4 h, resulted in partial transformation of austenite. As a result the carbon-supersaturated acicular ferrite, morphologically similar to bainitic ferrite was formed. The degree of this transformation increased with increasing concentrations of Cr and Mo, which successively decreased the thermodynamic stability of austenite. A change of matrix structure made it possible to significantly increase hardness and abrasive-wear resistance of castings. The largest increment of hardness and abrasion resistance was demonstrated by the castings with the highest total concentration of Cr and Mo with an addition of 0.4% Al. Introduction of Cr and Mo also resulted in an increase of corrosion resistance. In the heat-treated specimens, increasing the concentration of Cr and Mo resulted in a successive decrease of the depth of corrosion pits, with an increase in their number at the same time. This is very favourable from the viewpoint of corrosion resistance.

## 1. Introduction

A typical example of cast iron with relatively good machinability and corrosion resistance is Ni-Resist austenitic cast iron [1,2]. However, this is a material with low abrasion resistance. The alternative is Ni-Mn-Cu cast iron with a radically reduced Ni content compared to Ni-Resist cast iron, wherain the reduced Ni content is compensated by the addition of austenitizing elements, such as Mn and Cu [2].

Proper selection of chemical composition of the Ni-Mn-Cu cast iron makes it possible to obtain castings with an austenitic structure, guaranteeing good machinability [2]. In turn, proper selection of heat treatment parameters, provoking radical changes on the casting matrix, which makes it possible to obtain good mechanical properties and high abrasive-wear resistance while maintaining increased corrosion resistance (high electrochemical potential of the alloying elements) [3,4,5,6,7,8]. This makes it possible to use this type of cast iron to cast machine parts working under hard conditions such as those encountered in the mining industry.

Selection of chemical composition is mainly based on value of nickel equivalent Equ_Ni_, which indicates the thermodynamic stability of the austenitic matrix. If the equivalent value calculated according to Equation (1) [9] is smaller than 16.0%, it results in partial transformation of austenite to acicular ferrite [4,5,8,9].

Equ_Ni_ = 0.32⋅C + 0.13⋅Si + Ni + 2.48⋅Mn + 0.53⋅Cu (1)

where Equ_Ni_—nickel equivalent [wt %], C, Si, Ni, Mn, Cu—concentrations of elements [wt %].

The higher the degree of this transformation is, the smaller the nickel equivalent value [4,5,8]. This leads to significantly higher hardness of the castings, which considerably impedes their mechanical working.

In turn, if the Equ_Ni_ value is at least 16.0%, the matrix structure of raw castings is composed exclusively of austenite [9]. An increase of the equivalent value results in increased stability of the austenitic matrix [8,10]. This is a favourable phenomenon from the viewpoint of the possibility to obtain a structure durable in a wide range of temperatures. However, this restricts the possibility to obtain, by heat treatment, a hard and abrasion resistant structure, with respect to the properties similar to those of the austempered ductile (ADI) cast iron [11,12,13,14,15,16,17,18,19,20,21,22,23,24,25].

In this respect, it is most advantageous to use a cast iron with the Equ_Ni_ value of ca. 16.0%. This allows one to obtain raw castings with an austenitic matrix and to change this structure by a technically simple heat treatment (soaking and air-cooling) [6,7].

It seems possible to increase hardness, abrasive-wear resistance and corrosion resistance of Ni-Mn-Cu cast iron by introducing chromium and molybdenum, the elements commonly used to this end in other grades of cast iron. Due to the chilling effect of these elements, it seems reasonable to add, at the same time, a small amount of aluminium. Therefore, the purpose of the work was to determine to what extent additions of Cr, Mo and Al will affect the structure, hardness, abrasive-wear resistance and corrosion resistance of heat-treated castings.

## 2. Materials and Methods

Examinations were carried-out on cast iron coming from nine melts (Table 1). At the assumed constant concentration of basic elements: 3.4 ± 0.2% C; 1.8 ± 0.2% Si; 4.2 ± 0.2% Mn; 3.2 ± 0.2% Ni; 1.8 ± 0.2% Cu; 0.18 ± 0.2% P and 0.01% S, the concentrations of Cr and Mo were changed from 0.4 to 1.8% and from 0.2 to 0.5%, respectively. Moreover, an addition of 0.4% Al was applied in the alloys with higher concentrations of Cr and Mo, in order to compensate their chilling effect (tendency to create hard spots). Such a selection of chemical composition, developed on the basis of previous studies and literature data [26], should make it possible to obtain nearly-eutectic cast iron with low thermodynamic stability of the austenitic matrix of raw castings, and with limited tendency to create hard spots.

The cast iron was melted in an induction medium-frequency furnace, in a type A35 SiC crucible. Castings of alloys from *1* to *9* in the form of dia. 30 mm x 250 mm shafts were cast in shell moulds. Then, each of the obtained casts was cut into test samples in the form of 10 mm thick rollers, which were subjected to metallographic tests. Raw cast iron samples and after heat treatment were tested. Heat treatment of samples consisted in soaking at 500°C for 4 h (in a resistance furnace), followed by air cooling.

From the obtained castings, specimens were taken for chemical analysis, microscopic observations, hardness measurements, abrasive-wear resistance tests and corrosion resistance tests. All research results are average values from at least three measurements.

Chemical analysis was carried-out spectrally with use of a GDS 750 QDP glow discharge analyser (Leco, London, UK) and Quanta 250 scanning electron microscope (FEI, Waltham, MA, USA) equipped with an EDS detector. Results of the analysis, as well as values of nickel equivalent Equ_Ni_ (calculated according to the equation (1)) and of eutectic saturation ratio S_C_ (indicator of degree of deviation in the chemical composition of cast iron from its eutectic composition) are given in Table 1.

Microscopic examinations were performed using a MA200 light microscope (Nikon, Bangkok, Thailand) and TM 3000 (Hitachi, Tokyo, Japan) and FEI Quanta 250 scanning electron microscopes.

Brinell hardness was measured acc. to EN ISO 6506-1:2014-12 on an Nexus tester (Innovatest, Maastricht, Netherlands) with a ball dia. 2.5 mm under 1838.75 N. Vickers microhardness was measured acc. to EN ISO 6507-1:2018-05 on an Nova tester by Innovatest (Innovatest, Maastricht, Netherlands) under the indenter load of 0.01 N and 0.1 N.

Abrasive-wear resistance was determined by the „pin-on-disc” method on machine (Struers, Tokyo, Japan). Measurements consisted in abrading specimens dia. 25 mm pressed at 30 N against a diamond disk (grain size 45 to 53 µm) cooled with water. The measurements were performed in six cycles. Each cycle lasting 5 min corresponded to the sliding distance of 175 m. Therefore, each specimen was abraded for 30 min on the distance of 1050 m.

Corrosion resistance of the alloy was determined using the gravimetric and the potentiodynamic methods. In both cases, 3-% water solution of NaCl was used as the corrosive solution, at ambient temperature [27]. During gravimetric measurements, the corrosive medium was aerated in order to increase its aggressiveness [28].

Results of gravimetric tests are presented as mass loss per unit of time per unit area of the specimen V_C_ [mg/(dm^2^·day)] and, after conversion by the formula (2) [29,30], as linear corrosion rate V_P_:

V_P_ = (0.0365·V_C_)/d (2)

where V_P_—linear corrosion rate [mm/year], V_C_—mass loss of the specimen in time [mg/(dm^2^·day)], d—density of the metallic material [g/cm^3^].

Potentiodynamic measurements were carried-out in a completely automated three-electrode system, using a potentiostat (BioLogic, Seyssinet-Pariset, France). A saturated calomel electrode was used as the reference electrode. The auxiliary electrode was a platinum electrode [31,32,33]. Polarisation of all specimens was started from the potential of ca. -900 mV_NEC_, at 1 mV/s in the anodic direction. Corrosion resistance was determined on the grounds of cathodic-anodic transition potential E_K-A_, stationary potential E’, corrosion current density i_corr_ and polarisation resistance R_P_.

## 3. Results and Discussion

### 3.1. Microscopic Observations and Hardness Measurements of Raw Castings

**Figure 1:**
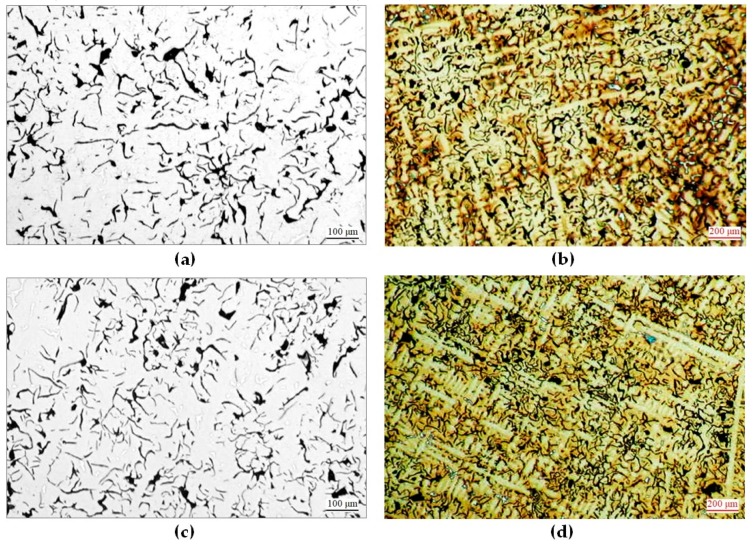

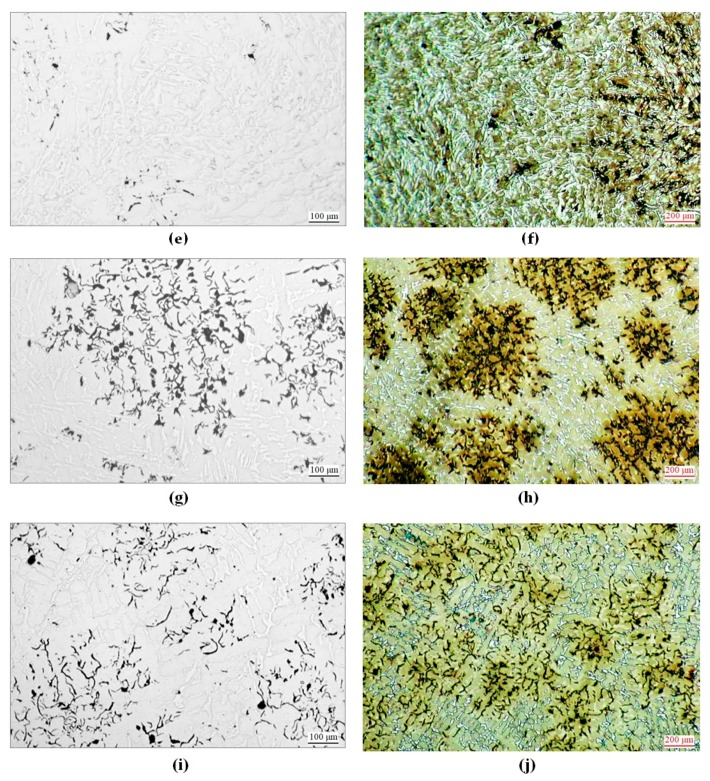
Microstructures of raw castings: (**a**) No. 1—graphite IA4; (**b**) No. 1—austenite, graphite; (**c**) No. 2—graphite IE4; (**d**)No. 2—austenite, graphite; (**e**) No. 5—graphite IE6; (**f**) No. 5—austenite, graphite, cementite; (**g**) No. 8—graphite ID5; (**h**) No. 8—austenite, graphite, cementite; (**i**) No. 9—graphite ID4; (**j**) No. 9—austenite, graphite, cementite. Polished sections on the left unetched; those on the right etched with Nital.

Microscopic observations, including determination of graphite features acc. to EN ISO 945-1:2018-04, were carried-out on polished sections unetched and etched with Nital, see Figure 1. Results of qualitative analysis of microstructure are given in Table 2.

Introduction of additional elements to Ni-Mn-Cu cast iron did not affect matrix structures of raw castings. In each case, the matrix was composed exclusively of austenite, see Figure 1b,d,f,h,j. However, features and quantities of graphite varied, see Figure 1a,c,e,g,i.

In the alloy No. 1 (with no addition of Cr and Mo), straight graphite type A size 4 was found, see Figure 1a. Introduction of 0.4% Cr to the alloy No. 2 resulted in a reduction of quantity and size of graphite particles. A tendency for interdendritic type E arrangement appeared, see Figure 1c. These tendencies increased along with increasing chromium concentration in successive alloys No. 3 to 5. Beginning from 0.9% Cr (the alloy No. 4 and next the alloy No. 5), in spite of introducing 0.4% Al, partial chilling appeared in the castings, see Figure 1f. An addition of molybdenum, like that of chromium, resulted in smaller quantity and size of graphite particles, and increased the inclination for chilling. However, this effect was less intensive than the influence of chromium. In consequence, the highest degree of chilling was found in the alloy No. 5 (the highest content of Cr) and No. 9 with the largest total content of both elements, see Figure 1f,j.

Introduction of additional elements and the related changes of microstructure resulted in changed HB hardness of the castings. The main factor deciding the hardness of raw castings was their chilling degree, strictly related to their total content of Cr and Mo. With increasing concentrations of these elements, the hardness of the alloys increased. A role was also played by HV hardness of austenite, which rose with increasing concentration of chromium: from 160 HV0.01N in the Cr-free alloy No. 1 to 230 HV0.01N in the alloy No. 5 with the highest concentration of Cr. In consequence, the lowest hardness (160 HBS) was demonstrated by the Cr-free alloy No. 1, and the highest hardness (380 HBS)—by the Al-free alloy No. 5 containing 1.8% Cr.

### 3.2. Microscopic Observations and Hardness Measurements of Heat-Treated Castings

**Figure 2:**
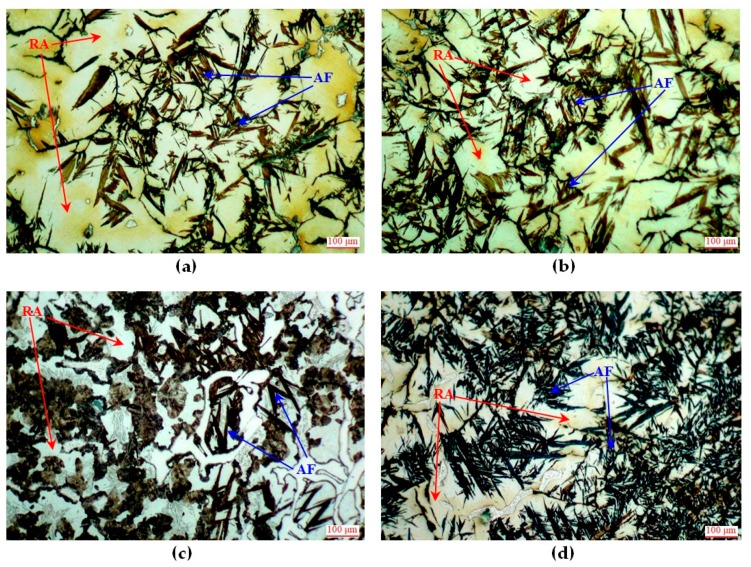

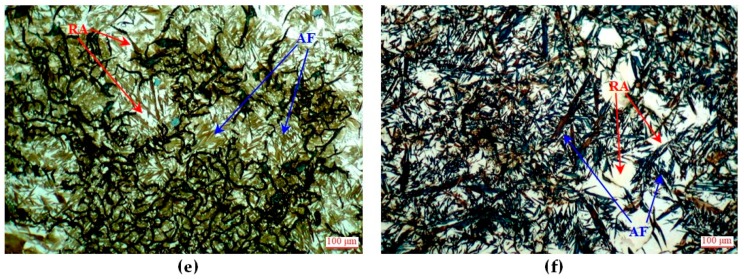
Microstructures of castings after heat treatment: (**a**) No. 1—retained austenite, acicular ferrite; (**b**) No. 2—retained austenite, acicular ferrite; (**c**) No. 5—retained austenite, acicular ferrite, pearlite; (**d**) No. 7– retained austenite, acicular ferrite; (**e**) No. 8—retained austenite, acicular ferrite; (**f**) No. 8—retained austenite, acicular ferrite. Etched with Nital. RA—retained austenite; AF—acicular ferrite.

Heat treatment (soaking at 500 °C for 4 h followed by air cooling) resulted in changes of matrix structures of all the castings. Austenite was partially transformed to acicular ferrite, morphologically comparable with ferrite present in upper bainite. The transformation degree was different in individual castings, see Figure 2 and Table 3.

The smallest changes occurred in the alloy No. 1 (with no Cr and Mo). The austenite transformation degree did not exceed 50%. Introducing and increasing concentrations of Cr and Mo successively increased this transformation degree. In the alloys No. 8 and No. 9 with the highest total concentration of both elements, from 85 to 90% of austenite underwent the transformation. This means that introduction of these elements reduced the thermodynamic stability of austenite.

Changes of matrix structure were accompanied by significant changes of hardness. Differences of hardness between individual castings were considered from two points of view: first—absolute hardness and second—hardness increment caused by heat treatment.

The lowest hardness (313 HBW) was found for the alloy No. 1 with the lowest austenite transformation degree. Higher hardness of the other alloys resulted from their increased inclination to chilling and/or increased degree of austenite transformation. Among the alloys with similar initial structure, i.e., the alloys No. 2, 3 and No. 6, 7 higher hardness after heat treatment was demonstrated by the castings with higher concentrations of chromium. The highest hardness (492 HBW) was obtained for the alloy No. 5 containing 1.8% Cr. The main cause of so high hardness of this alloy was very strong chilling of raw castings, meaning also their poor machinability.

From the viewpoint of the possibility to obtain castings with good machinability and high hardness, the alloy should be characterised by low inclination to chilling and low stability of austenite. An example is the alloy No. 8, whose hardness of the raw casting was 210 HBS. After heat treatment, its hardness was over twice higher, reaching 441 HBW. This resulted from very high degree of austenite transformation (ca. 90%) to carbon-supersaturated acicular ferrite. Concentration of carbon in this ferrite was 0.33 ± 0.04 %C and its hardness was 510 to 550 HV0.1N.

### 3.3. Abrasive-Wear Resistance Testing

The heat-treated castings were subjected to abrasive-wear resistance tests. Results are presented in form of the wear rate coefficient, see Table 4. The obtained results indicate occurrence of a strong relation between abrasive-wear resistance of cast iron and its chilling degree, austenite transformation degree and hardness.

The highest wear rate, indicating the lowest abrasive-wear resistance, was demonstrated by the chromium-free alloy No. 1, free from hard spots and with the lowest austenite transformation degree. A lower wear rate of the other alloys resulted from increased chilling degree of the castings and/or increased austenite transformation degree. For this reason, among the alloys with comparable initial structure, e.g., the alloys No. 2 and 3 or the alloys No. 6 and 7, lower wear rate was demonstrated by the castings with higher concentration of chromium. The lowest wear rate was shown by the almost completely chilled alloy No. 5 containing 1.8% Cr. However, the most favourable solution, from the viewpoint of the possibility to obtain castings with good machinability and high resistance to abrasive wear, appeared the alloy No. 8 (with no hard spots and with a very high degree of austenite transformation). Its wear rate was similar to that of the alloy No. 5.

After abrasive-wear testing, the specimens were subjected to observation of their surface topography, using the SEM imaging technique. Smaller topographic diversification of the surface, indicating uniform wear, can suggest higher resistance to abrasion. Values of the indices determining surface topography were determined using a scanning electron microscope. Individual indices were determined on measuring lengths of ca. 30 mm. The following average profile parameters were determined: the average highest peak (R_pAVR_), the average lowest valley (R_vAVR_) and the average distance between these two values (R_zAVR_ = R_pAVR_ + R_vAVR_), the average arithmetic deviation of the profile from the average line measured along the testing section (R_aAVR_) and the average square profile deviation from the average line measured along the testing section (R_qAVR_). Results are given in Table 4. The smallest topographic diversification was shown by the alloys No. 5 and No. 8. In the alloy No. 5 it was caused by almost complete chilling (ca. 95%), but in the alloy No. 8 it resulted from very high degree of austenite transformation (ca. 90%).

### 3.4. Corrosion Resistance Testing

In order to obtain reliable results of corrosion-resistance testing, two research methods were applied: the gravimetric method and the potentiodynamic method.

Gravimetric measurements were continued for 24 days. The specimens were weighed (after cleaning) after the following times of exposure in 3-% water solution of NaCl: 1, 2, 5, 8, 13, 18 and 24 days. Corrosion rates in function of time were determined according to the formula (2). Results are presented in Table 5.

Gravimetric examinations revealed slight differences in corrosion resistance between individual alloys. After 1 day of maintaining the specimens in the corrosive solution, corrosion rate of raw castings ranged between 0.49 and 0.55 mm/year. Its largest value was measured for the alloy No. 1 (containing no Cr) and the smallest value—for the chilled alloy No. 5 (containing 1.8% Cr). Extension of the exposure time to 2 days resulted in higher corrosion rate of all the alloys. Maintaining the specimens for 2 to 5 days resulted in successive reduction of corrosion rate of all the alloys. This phenomenon is very favourable from the viewpoint of corrosion resistance. After 24 days of exposure, reduction of corrosion rate by ca. 30 to 40% in relation to the initial values was found in all cases.

The corrosion rate of the castings after heat treatment was slightly lower in comparison to that of raw castings. After 1 day of exposure, corrosion rate of heat-treated specimens ranged between 0.50 and 0.57 mm/year. Like for raw castings, its highest value was found for the Cr-free alloy No. 1, but the lowest value was found for the chilled alloy No. 5 with the highest concentration of Cr.

From the viewpoint of the possibility to obtain castings resistant to both abrasive wear and corrosion, the most favourable features were demonstrated by the alloy No. 8 containing Cr, Mo and Al. Corrosion rate of this alloy was similar to that of the most corrosion-resistant alloy No. 5. The element that most effectively increased corrosion resistance, appeared chromium.

Soaking reduces segregation of elements and can also reduce depth of corrosion pits [10], so the potentiodynamic tests were carried-out only on the heat-treated alloys that demonstrated increased resistance to abrasive wear.

During potentiodynamic tests, the specimens were subjected to polarisation after 30 min and after 48 h of their keeping in 3-% water solution of NaCl. Results are given in Table 6.

The largest values of stationary potential E’, indicating elevated corrosion resistance, were found for the alloy No. 1 both after 30 min (−565 mV) and after 48 h (−524 mV), see Table 6. This alloy showed the lowest transformation degree of its austenitic matrix, stabilised by the largest total content of Ni, Mn and Cr (Equ_Ni_ = 16.3%). The smallest E’ value was found for the alloy No. 9 (−605 mV after 30 min and −558 mV after 48 h). This is the alloy with the highest austenite transformation degree. Apart from Ni, Mn and Cu, it contained also additions of Cr, Mo and Al. Longer exposure time resulted in larger E’ values of all the specimens. This is favourable from the viewpoint of corrosion resistance of the examined alloys.

**Figure 3:**
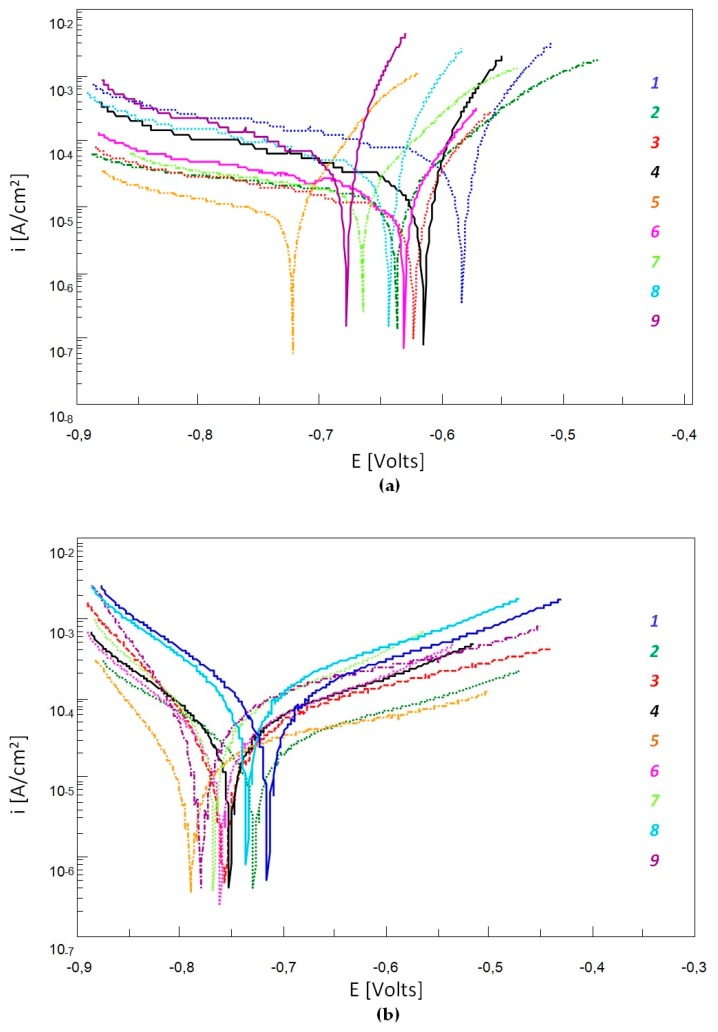
Polarisation curve for the alloys No. 1 to No. 9 after 30-min (**a**) and 48-h (**b**) exposure in 3-% aqueous solution of NaCl.

Relatively big differences between values of the cathodic-anodic transition potential E_K-A_ (ca. 144 mV after 30-min exposure) for the alloys No. *1* and No. *5* indicate a diversity of electrode processes occurring on the metallic surface, see Table 6 and Figure 3. This is related to the phase composition diversity of the examined alloys. Longer time of exposure in the corrosive solution resulted in smaller differences between E_K-A_ values of individual alloys.

Values of corrosion current density i_corr_ and of polarisation resistance R_p_ showed the inversely proportional relation, see Table 6. Smaller i_corr_ values and larger R_p_ values often indicate increased corrosion resistance. However, the obtained results do not indicate radical differences of these values between individual alloys (Table 6 and Figure 3). After 30-min exposure, the i_corr_ values ranged from 18.6 to 23.2 μA/cm^2^, but the R_p_ values ranged within 1.2 to 1.6 kΩ·cm^2^. In turn, lower exposure time resulted in slightly increased i_corr_ values (79.5 to 98.8 μA/cm^2^)and decreased R_p_ values (0.2 to 0.5 kΩ·cm^2^).

The results of potentiodynamic tests did not show radical differences between the corrosion resistances of individual alloys. Longer exposure time of specimens did not cause significant changes of their corrosion resistance, either. This is why additional observations of surface topography of the specimens after potentiodynamic testing and after 48-h exposure in the corrosive solution were carried out. Results are given in Table 7. 

**Figure 4:**
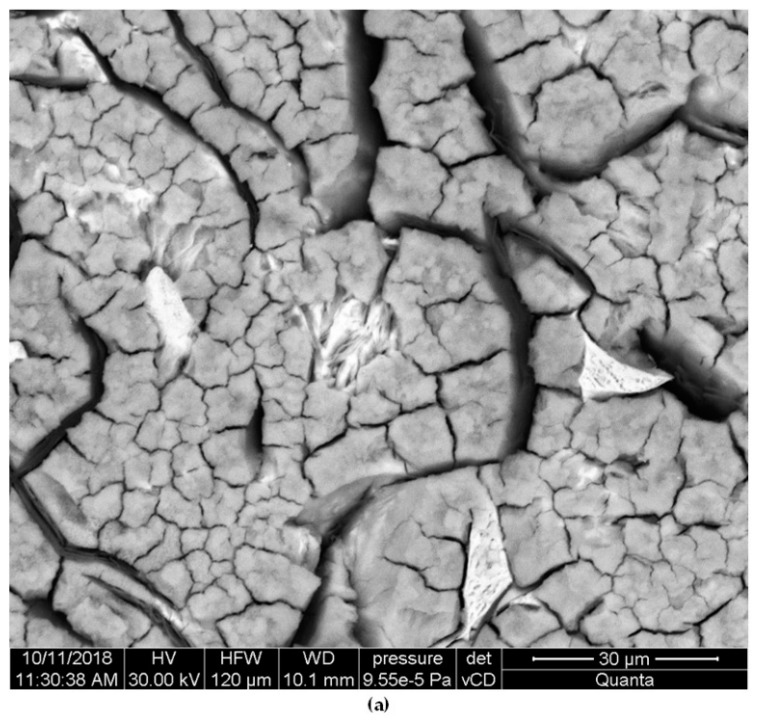

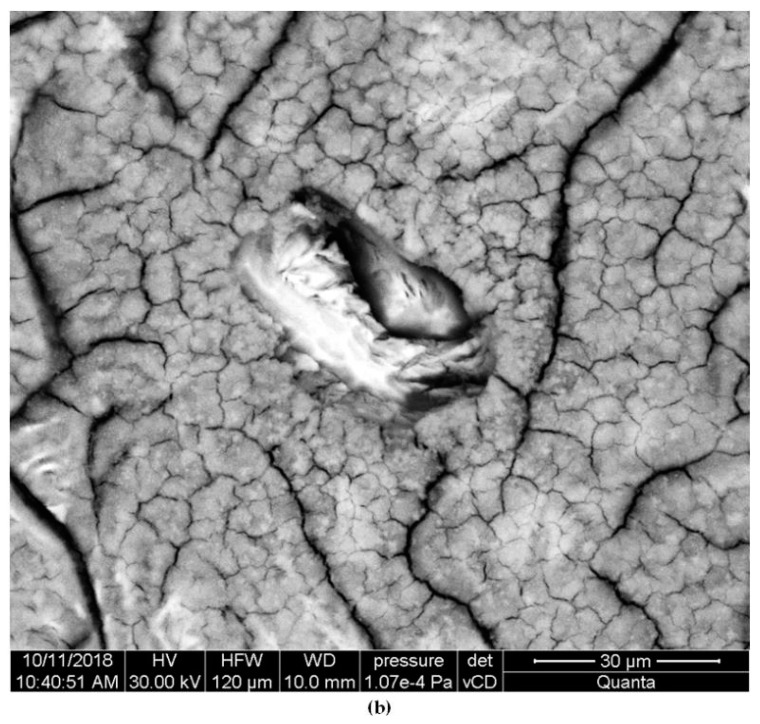
Face surfaces of specimens after potentiodynamic tests, exposed previously for 48 h in 3-% solution of NaCl: alloy No. 1 (**a**) and alloy No. 8 (**b**). Unetched.

**Figure 5:**
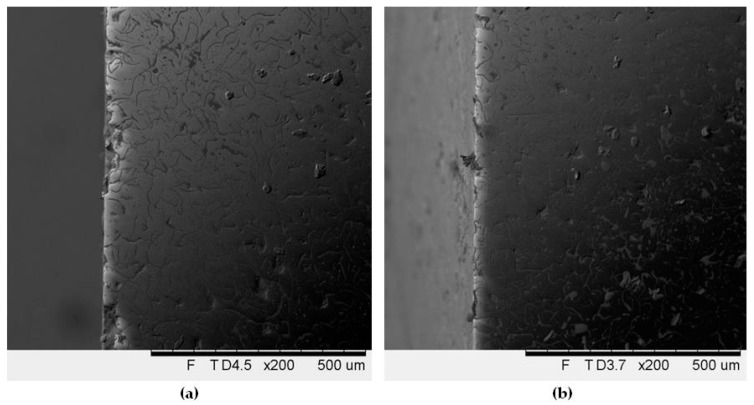
Cross-section surfaces of specimens after potentiodynamic tests, exposed previously for 48 h in 3-% solution of NaCl: alloy No. 1 (**a**) and alloy No. 8 (**b**). Unetched.

As for the differences between individual samples, in the alloy No. 1 with the largest Equ_Ni_ value, a small number of relatively deep pits were found. Introduction of Cr, also Mo (that reduces stability of austenitic matrix) resulted in successive decrease of depth of the pits accompanied by an increase of their number. This effect is favourable from the viewpoint of corrosion resistance. The largest number of pits with the smallest depth was found in the alloys with the high total content of Cr, Mo and Al, the highest degree of austenite transformation and fragmentation of eutectic colonies. In all the alloys, corrosion damages were located mainly in vicinity of eutectic colonies and of phase boundaries, see Figure 4 and Figure 5. This was due to differences in electrochemical potentials between individual phases.

**Figure 6:**
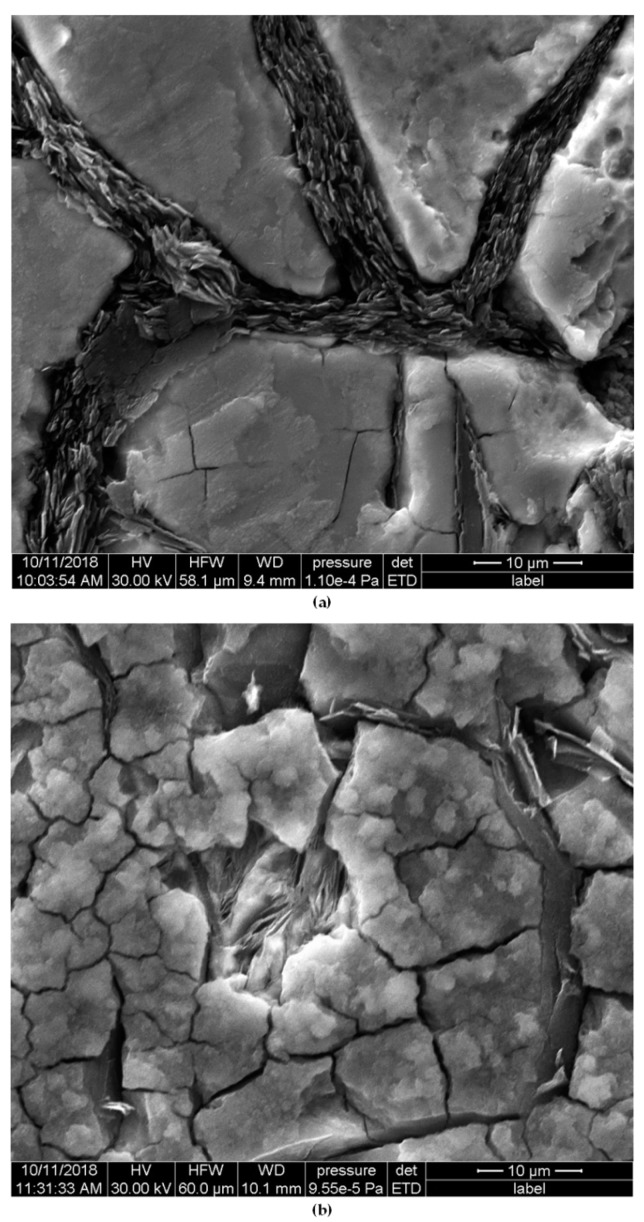
Face surfaces of specimens alloy No. 1 after potentiodynamic tests, exposed previously in 3-% solution of NaCl for: 30 min (**a**) and 48h (**b**). Unetched.

The mechanism of corrosion damage was typical for pitting corrosion. He was discussed on example of alloy No. 1, due to the smallest degree of austenite conversion, and thus the readability of analysis. After 30 min exposure of samples in corrosive solution, the greatest corrosion damage was observed primarily near graphite flakes (Figure 6a) This was due to significant differences of electrochemical potentials between graphite (+0.372 V) and the cast iron matrix (−0.776 V) [26,27]. As a result of a large potential difference, which was about 1V, micro-cells were formed, increasing the corrosion rate. After the samples were stored in a corrosive solution for 48 h, deepening of pits near the graphite was found. In addition, corrosion damage appeared in the matrix, mainly at the boundaries of austenite grains (Figure 6b).

## 4. Conclusions

The matrix structure of all raw castings was composed exclusively of austenite. The accepted concentration ranges of Cr, Mo and Al did not cause the phase transformations that occur in the case of too low thermodynamic stability of austenite. However, it was found that hardness of austenite increased with increasing concentrations of Cr and Mo.

As the contents of mainly Cr and Mo increased, the quantity and size of graphite particles decreased and a tendency to their interdendritic arrangement increased. These elements resulted in higher susceptibility of the alloy to chilling (creating hard spots). This resulted in a clearly higher hardness of raw castings. An addition of 0.4% Al partially restricted this tendency.

Heat treatment led to partial austenite transformation to carbon-supersaturated, hard acicular ferrite in all castings. The transformation degree increased with increasing concentrations of Cr and Mo, which is an evidence that both elements reduce the thermodynamic stability of the austenitic matrix.

The largest increments of hardness and abrasive-wear resistance, caused by heat treatment, occurred in the castings with the highest degree of austenite transformation, that is in the castings with the largest content of Cr and Mo, and with an addition of 0.4% Al.

At the same time, introduction of Cr and Mo to cast iron (elements with relatively high electrochemical potential) resulted in increased corrosion resistance. Increasing concentrations of Cr and Mo (mainly Cr) resulted in lower corrosion rates of raw castings because as the total content of both elements increased, the quantity and size of graphite flakes decreased (usually damages were found near them), the tendency to interdendritic distribution and fragmentation of eutectic colonies increased. Heat treatment of the castings resulted in unimportant, in comparison to increased abrasion resistance, reduction of the corrosion resistance in relation to raw castings. Introduction of Cr and Mo, the elements reducing the stability of the austenitic matrix, resulted in a successive reduction of the depth of corrosion pits, accompanied by an increase in their number. From the viewpoint of corrosion resistance, this phenomenon is desirable.

## Figures and Tables

**Table 1:** Chemical composition of raw casts, nickel equivalent Equ_Ni_ and eutectic saturation ratio S_C._

*Alloy* *No.*	Chemical Composition [wt %]										S_C_ [ / ]	Equ_Ni_ [wt %]
	C	Si	Mn	Ni	Cu	Cr	Mo	Al	P	S		
1	3.5	1.9	4.3	3.3	1.9	−	−	−	0.19	0.01	1.02	16.3
2	3.6	1.8	4.2	3.2	2.0	0.4	−	−	0.19	0.01	1.03	16.1
3	3.4	1.7	4.4	2.9	1.7	0.7	−	−	0.20	0.01	0.95	16.0
4	3.5	1.8	4.2	3.4	1.8	0.9	−	0.4	0.18	0.01	1.02	16.1
5	3.4	2.0	4.1	3.4	2.0	1.8	−	0.4	0.17	0.01	0.99	16.0
6	3.4	2.0	4.3	3.3	1.7	0.7	0.2	−	0.18	0.01	0.97	16.2
7	3.6	1.9	4.3	3.1	1.8	1.0	0.2	−	0.16	0.01	1.01	16.1
8	3.3	1.7	4.2	3.3	1.9	0.7	0.5	0.4	0.20	0.01	0.95	16.0
9	3.5	1.8	4.2	3.2	2.0	1.1	0.5	0.4	0.18	0.01	1.01	16.0

**Table 2:** Composition of microstructure and hardness of raw castings.

*Alloy No.*	Equ_Ni_ [wt%]	Matrix	High-Carbon Phases %Fe_3_C – %C_gr_* /type of C_gr_/	HBS_AVR_ 2.5/187.5kG [/] (+/−2)	HV0.01N_AVR_ of Austenite [/] (+/−2)
1	16.3	austenite	0% Fe_3_C – 100%C_gr_ /IA4/	160	168
2	16.1	austenite	0% Fe_3_C – 100%C_gr_ /IE4/	170	182
3	16.0	austenite	0% Fe_3_C – 100%C_gr_ /IE4/	185	192
4	16.1	austenite	10% Fe_3_C – 90%C_gr_ /IE5/	280	204
5	16.0	austenite	95% Fe_3_C – 5%C_gr_ /IE6/	380	230
6	16.2	austenite	45% Fe_3_C – 55%C_gr_ /ID5/	205	195
7	16.1	austenite	50% Fe_3_C – 50%C_gr_ /ID5/	220	203
8	16.0	austenite	55% Fe_3_C – 45%C_gr_ /ID5/	210	214
9	16.0	austenite	60% Fe_3_C – 40%C_gr_ /ID4/	250	207

* Fe_3_C—cementite; C_gr_—graphite; Fe_3_C + C_gr_ = 100%high-carbon phases.

**Table 3:** Composition of microstructure and hardness for heat-treated castings.

*Alloy* *No.*	Equ_Ni_ [wt %]	Matrix Components * A – Fe_m_ – P [%– % –%]	Form of Carbon in Eutectic Mixture **	HBW_AVR_ 2.5/187.5 [/] (+/−3)	Increase of HBW 2.5/187.5 [/]
1	16.3	50 – 50 – 0	C_gr_	313	154
2	16.1	48 – 52 – 0	C_gr_	347	178
3	16.0	47 – 53 – 0	C_gr_	372	189
4	16.1	45 – 55 – 0	C_gr +_ Fe_3_C	411	129
5	16.0	45 – 25 – 30	C_gr +_ Fe_3_C	492	112
6	16.2	45 – 55 – 0	C_gr +_ Fe_3_C	362	157
7	16.1	40 – 60 – 0	C_gr +_ Fe_3_C	383	163
8	16.0	10 – 90 – 0	C_gr +_ Fe_3_C	441	230
9	16.0	15 – 85 – 0	C_gr +_ Fe_3_C	454	205

* A—austenite; Fe_m_—acicular ferrite; P—pearlite. ** Fe_3_C—cementite; C_gr_—graphite; Fe_3_C + C_gr_ = 100% high-carbon phases.

**Table 4:** Increase of hardness and abrasive-wear indicators for heat-treated castings.

*Alloy* *No.*	Increase of HBW 2.5/187.5 [/]	Wear Rate [mg/m∙10^4^]	Decrease in Wear Rate Compared to Alloy No. 1	Index of Surface Topography [μm]				
				R_pAVR_ (+/−0.02)	R_vAVR_ (+/−0.02)	R_zAVR_ (+/−0.02)	R_aAVR_ (+/−0.02)	R_qAVR_ (+/−0.02)
1	154	2.14	−	5.17	12.19	17.36	0.87	1.44
2	178	1.62	−0.57	4.18	10.88	15.06	0.54	0.85
3	189	1.52	−0.67	4.19	10.83	15.02	0.56	0.87
4	129	1.24	−0.95	4.16	10.84	15.00	0.52	0.85
5	112	0.57	−1.62	4.01	10.02	14.03	0.48	0.83
6	157	1.14	−1.05	4.17	10.78	14.95	0.50	0.84
7	163	1.05	−1.14	4.14	10.74	14.88	0.49	0.83
8	230	0.85	−1.33	4.05	10.53	14.58	0.45	0.81
9	205	1.62	−1.29	4.09	10.68	14.77	0.44	0.84

* A—austenite, Fe_m_—acicular ferrite, P—pearlite. ** Fe_3_C—cementite; C_gr_—graphite; Fe_3_C + C_gr_ = 100% high-carbon phases.

**Table 5:** Corrosion rate V_P_ after exposure of specimens in 3-% solution of NaCl.

*Alloy* *No.*		V_P_ [mm/year] After Exposure for Specified Time (days)						
		1	2	5	8	13	18	24
1	as cast	0.55	0.58	0.56	0.52	0.48	0.44	0.39
	heat-treated	0.57	0.59	0.57	0.55	0.51	0.45	0.37
2	as cast	0.55	0.57	0.54	0.51	0.46	0.42	0.37
	heat-treated	0.56	0.59	0.55	0.53	0.47	0.44	0.38
3	as cast	0.53	0.55	0.54	0.50	0.44	0.42	0.35
	heat-treated	0.53	0.56	0.55	0.53	0.44	0.43	0.37
4	as cast	0.52	0.53	0.52	0.51	0.43	0.43	0.33
	heat-treated	0.53	0.55	0.53	0.52	0.43	0.45	0.34
5	as cast	0.49	0.51	0.50	0.48	0.44	0.41	0.30
	heat-treated	0.50	0.52	0.50	0.48	0.46	0.42	0.31
6	as cast	0.53	0.54	0.54	0.51	0.43	0.42	0.35
	heat-treated	0.54	0.56	0.54	0.52	0.42	0.43	0.36
7	as cast	0.52	0.53	0.52	0.50	0.42	0.41	0.33
	heat-treated	0.53	0.54	0.53	0.52	0.41	0.42	0.34
8	as cast	0.51	0.53	0.50	0.49	0.42	0.41	0.31
	heat-treated	0.52	0.53	0.51	0.50	0.41	0.43	0.31
9	as cast	0.50	0.51	0.50	0.49	0.43	0.42	0.30
	heat-treated	0.50	0.52	0.50	0.51	0.41	0.42	0.31

**Table 6:** Electrochemical indicators characterising corrosion process.

*Alloy No.*	E′ [mV]		E_K-A_ [mV]		i_corr_ [μA/cm^2^]		R_P_ [kΩ·cm^2^]	
	30 min	48 h	30 min	48 h	30 min	48 h	30 min	48 h
1	−565	−524	−582	−733	23.2	98.8	1.2	0.3
2	−569	−535	−643	−739	20.1	92.3	1.2	0.2
3	−572	−538	−621	−753	19.3	89.7	1.3	0.3
4	−581	−544	−618	−751	18.7	84.5	1.3	0.3
5	−589	−548	−726	−790	18.9	79.5	1.5	0.5
6	−568	−533	−635	−758	19.8	94.0	1.3	0.3
7	−572	−539	−669	−773	19.8	88.5	1.4	0.3
8	−597	−556	−659	−790	18.6	79.5	1.6	0.5
9	−625	−588	−678	−785	20.2	94.3	1.4	0.3

**Table 7:** Indicators describing surface topography of specimens after potentiodynamic tests (after 48-h exposure in 3-% aqueous solution of NaCl).

*Alloy* *No.*	Equ_Ni_ [wt %]	Cr [wt %]	Mo [wt %]	Al [wt %]	Surface Topography Index [μm]		
					R_pAVR_ (+/−0.03)	R_vAVR_ (+/−0.03)	R_zAVR_ (+/−0.03)
1	16.3	−	−	−	5.01	34.64	39.65
2	16.1	0.4	−	−	4.98	33.35	38.33
3	16.0	0.7	−	−	4.85	32.21	37.06
4	16.1	0.9	−	0.4	4.77	28.25	33.02
5	16.0	1.8	−	0.4	4.73	21.14	25.87
6	16.2	0.7	0.2	−	4.86	24.17	29.03
7	16.1	1.0	0.2	−	4.72	22.14	28.86
8	16.0	0.7	0.5	0.4	4.61	19.08	23.75
9	16.0	1.1	0.5	0.4	4.58	19.14	23.66
